# Characterization of Behavioral, Neuropathological, Brain Metabolic and Key Molecular Changes in zQ175 Knock-In Mouse Model of Huntington’s Disease

**DOI:** 10.1371/journal.pone.0148839

**Published:** 2016-02-09

**Authors:** Qi Peng, Bin Wu, Mali Jiang, Jing Jin, Zhipeng Hou, Jennifer Zheng, Jiangyang Zhang, Wenzhen Duan

**Affiliations:** 1 Division of Neurobiology, Department of Psychiatry and Behavioral Sciences, Johns Hopkins University School of Medicine, Baltimore, Maryland, United States of America; 2 Department of General Practice, The First hospital of China Medical University, Shenyang, Liaoning Province, China; 3 Department of Radiology, Johns Hopkins University School of Medicine, Baltimore, Maryland, United States of America; 4 Department of Neuroscience, Johns Hopkins University School of Medicine, Baltimore, Maryland, United States of America; 5 Program in Cellular and Molecular Medicine, Johns Hopkins University School of Medicine, Baltimore, Maryland, United States of America; Grenoble Institut des Neurosciences, Universite Grenoble Alpes, FRANCE

## Abstract

Huntington’s disease (HD) is caused by an expansion of the trinucleotide poly (CAG) tract located in exon 1 of the *huntingtin* (*Htt*) gene leading to progressive neurodegeneration in selected brain regions, and associated functional impairments in motor, cognitive, and psychiatric domains. Since the discovery of the gene mutation that causes the disease, mouse models have been developed by different strategies. Recently, a new model, the zQ175 knock-in (KI) line, was developed in an attempt to have the *Htt* gene in a context and causing a phenotype that more closely mimics HD in humans. The behavioral phenotype was characterized across the independent laboratories and important features reminiscent of human HD are observed in zQ175 mice. In the current study, we characterized the zQ175 model housed in an academic laboratory under reversed dark-light cycle, including motor function, *in vivo* longitudinal structural MRI imaging for brain volume, MRS for striatal metabolites, neuropathology, as well as a panel of key disease marker proteins in the striatum at different ages. Our results suggest that homozygous zQ175 mice exhibited significant brain atrophy before the motor deficits and brain metabolite changes. Altered striatal medium spiny neuronal marker, postsynaptic marker protein and complement component C1qC also characterized zQ175 mice. Our results confirmed that the zQ175 KI model is valuable in understanding of HD-like pathophysiology and evaluation of potential therapeutics. Our data also provide suggestions to select appropriate outcome measurements in preclinical studies using the zQ175 mice.

## Introduction

Huntington’s disease (HD) is a progressive, inherited neurodegenerative disorder characterized by involuntary movements, cognitive impairment, and psychiatric manifestations. The average onset of symptoms typically occurs in midlife, although it can range from early childhood (Juvenile form) to over 70 years of age. Of all the neurodegenerative disorders, HD possibly holds the greatest promise in the search for a disease-modifying therapy because HD is caused by a single gene mutation, in contrast to other neurodegenerative disorders in which the precise cause and pathogenic mechanisms are less well understood. Nonetheless, to date, proven neuroprotective strategies remain elusive, although there has been rapid progress in the understanding of the pathogenic mechanisms and development of novel therapeutic strategies. Part of the problem has been that the model system we used to develop therapeutics does not fully reflect human genetics and disease phenotype, and most of the trials to date have attempted intervening at a time when the degenerative process is already far advanced, when it would be difficult for even the most effective therapy to demonstrate any benefit. Through genetic testing, people who will ultimately develop HD can be identified years before clinical onset, raising the possibility of initiating therapy in the prodromal period to delay or prevent disease onset. In order to predict the effectiveness of disease-modifying therapies and prepare candidate treatment for clinical trials, appropriate animal models and molecular markers that reflect neuronal dysfunction have become of paramount importance.

Since the mutation responsible for the disease was identified in 1993, numerous mouse models of HD have been generated to study disease pathogenesis and evaluate potential therapeutic approaches. Of these, full-length Htt knock-in (KI) models best mimic the human disease condition from a genetic perspective, since the expression of the mutant *Htt* occurs in the appropriate genetic and protein context. However, most KI mouse models in which the expanded CAG repeat is inserted into the mouse *htt* typically show more subtle behavioral, histopathological, and molecular phenotypes compared to the transgenic models that overexpress mutant *htt* [1]. The zQ175 KI mouse, derived from a spontaneous expansion of the CAG copy number in the CAG 140 knock-in colony, exhibited clear behavioral deficits in both heterozygous and homozygous mice, especially in the dark phase of the diurnal cycle [2]. Decreased body weight, motor deficits, brain atrophy, altered brain metabolites, and decrease of striatal gene markers were also observed in the zQ175 model [2, 3].

We present the results of longitudinal characterization of behavioral, neuropathological, metabolic, and molecular changes in the zQ175 KI line under dark-light reversed housing condition in an academic laboratory. Mutant Htt aggregates were detected to be widely distributed in different brain regions. Altered striatal medium spiny neuronal marker protein, postsynaptic marker, and a complement component protein were evident at different disease stages in zQ175 mouse striatum. Our work proves the usefulness of this model to further investigate biological pathways affected by mutant *Htt* and to evaluate interventions to modify both disease onset and progression.

## Materials and Methods

### Mice

Sixteen homozygous zQ175 mice (8 males and 8 females) and 16 wild-type littermate controls (WT, 8 males and 8 females) were acquired from Jackson Lab (Bar Harbor, ME) for longitudinal characterization. In addition, 30 zQ175 mice and 30 WT littermate controls were used for molecular characterization (10 mice per group for each age, 5 males and 5 females), and 10 WT and 10 zQ175 male mice were used for histological study. zQ175 mice, originating from the CAG 140 mice, were generated by Psychogenics Inc. and the line is maintained in the Jackson Laboratories. Genotyping and CAG repeat count were determined at Laragen Inc. (Culver City, CA, USA) by PCR of tail snips. The CAG repeat length was 176 ± 8 in homozygous zQ175 mice used in the study. All mice were housed at 5 mice per cage under specific pathogen-free conditions with a reversed 12-h light/dark cycle maintained at 23°C, and provided with food and water ad libitum. All behavioral tests and longitudinal measures were done in the dark phase. This study was carried out in strict accordance with the recommendations in the Guide for the Care and Use of Laboratory Animals of the National Institutes of Health and approved by Institutional Animal Care and Use Committee of Johns Hopkins University. The protocol was approved by the Committee on the Ethics of Animal Care and Use Committee (Permit Number: MO15M176). All procedures were performed under isoflurane anesthesia, and all efforts were made to minimize suffering.

### Balance Beam Test

Motor function was assessed on an 80-cm long and 5-mm wide square-shaped or 11-mm diameter round-shaped balance beam that was mounted on supports of 50-cm in height. A bright light illuminated the start platform, and a darkened enclosed 1728 cm^3^ escape box (12 × 12 × 12 cm) was situated at the end of the beam. Disposable pads placed under the beam provided cushioning if an animal fell off the beam. Mice were trained to walk across the beam twice at least 1 h prior to testing. If a mouse stopped during training, the tail was gently pressed to encourage movement. After the training trial, mice were left undisturbed for at least an hour before testing. The time for each mouse to traverse the balance beam was recorded with a 125-sec maximum cut-off, and falls were scored as 125 sec.

### Open Field Locomotor Activity Test

Open field testing was performed during the dark phase of the diurnal cycle under red light conditions. The open field test was performed at the age of 3 months, 6 months, 9 months and 12 months of age. The mice were housed in the experimental room. The locomotor activity was measured by automated Open Field Activity System and the data were analyzed by Activity Monitor software (Columbus Instrument Inc., OH). The activity chambers (27.3 cm×27.3 cm×20.3 cm) were equipped with infrared beams. Mice were placed in the center of the chamber and their behavior was recorded for 60 min in 5-min bins for peripheral and central activity as well as rear frequency.

### Rotarod Test

Mice were tested for three consecutive days. Each daily session included a single training trial of 5 min at 4 RPM on the rotarod apparatus (Rotamex, Columbus Instrument, OH). One hour later, the mice were tested for 3 consecutive accelerating trials of 5 min with the rotarod speed changing from 4 to 40 RPM over 5 min, with an inter-trial interval of at least 30 min. The latency to fall from the rod was recorded for each trial and data are presented as average from three trials and three days, with mice remaining on the rod for more than 5 min removed and scored at 300 s.

### *In Vivo* Structural MRI Acquisition

*In vivo* MRI was performed on a vertical 9.4 Tesla MR scanner (Bruker Biospin, Billerica, MA, USA) with a triple-axis gradient and a physiological monitoring system (EKG, respiration, and body temperature). Mice were anesthetized with isoflurane (1%) mixed with oxygen and air at 1:3 ratios via a vaporizer and a facial mask and scanned longitudinally (the same mice were imaged repeatedly over a 12-month period). We used a 20-mm diameter volume coil as the radiofrequency transmitter and receiver. Temperature was maintained by a heating block built into the gradient system. Respiration was monitored throughout the entire scan. High-resolution anatomical images were acquired by using a three-dimensional (3D) T2-weighted fast spin echo sequence with the following parameters: echo time (TE)/repetition time (TR) = 40/700 ms, resolution = 0.1 mm × 0.1 mm × 0.25 mm, echo train length = 4, number of average = 2, and flip angle = 40°. Multi-slice T2-weighted images of the mouse brain were acquired by the RARE (Rapid Acquisition with Refocused Echoes) sequence with the following parameter (echo time (TE) / repetition time (TR) = 40 ms/1500 ms, RARE factor = 8, in-plane resolution = 0.125 mm x 0.125 mm, slice thickness = 1 mm, total imaging time less than 2 min) and used for planning the MRS voxel position and high resolution anatomical imaging. Total imaging time was about 50 min per mouse. Mice recovered quickly once the anesthesia was turned off, and all mice survived the imaging sessions.

### Structural MRI Image Analysis

Images were first rigidly aligned to a template image by using automated image registration software (http://bishopw.loni.ucla.edu/AIR5/, AIR). The template image was selected from one of the images acquired from age-matched littermate control mice (mouse had the medium brain volume among the control group), which had been manually adjusted to the orientation defined by the Paxinos atlas with an isotropic resolution of 0.1 mm x 0.1 mm x 0.1 mm per pixel. After rigid alignment, images had the same position and orientation as the template image, and image resolution was also adjusted to an isotropic resolution of 0.1 mm × 0.1 mm × 0.1 mm per pixel. Signals from non-brain tissue were removed manually (skull-stripping). Skull-stripped, rigidly aligned images were analyzed by using Landmarker software (www.mristudio.org). Intensity values of the gray matter, white matter, and cerebral spinal fluid were normalized to the values in the template images by using a piece-wise linear function. This procedure ensured that subject image and template image have similar intensity histograms. The intensity-normalized images were submitted by Landmarker software to a linux cluster, which runs Large Deformation Diffeomorphic Metric Mapping (LDDMM). The transformations were then used for quantitative measurement of changes in local tissue volume among different mouse brains, by computing the Jacobian values of the transformations generated by LDDMM.

### *In Vivo* MRS Acquisition and Data Analysis

Longitudinal MRS was conducted in the same cohort of mice for MRI study. Localized proton spectra were acquired by use of a Point Resolved Spectroscopy (PRESS) pulse sequence on the 9.4 Tesla MR scanner after structural MRI with the following parameters: TE/TR = 8.8 ms / 3000 ms. A 3 ×3 ×3 mm^3^ voxel was placed in the frontal forebrain and covered the striatum. An unsuppressed water reference scan was acquired with two signal averages for quantification of the proton peak, and water suppressed signals were acquired with 1024 signal averages for quantification of other metabolites. Quantification of MRS spectra was performed by using the LCModel [4, 5] with unsuppressed water as internal reference. Specifically, pulse sequence parameters and molecular structure were used to generate basis sets for each metabolite. Then metabolites as well as water signals were fitted to simulated basis sets to acquire the resonance amplitudes, after which the metabolite concentrations were estimated by referencing to known water concentrations. Metabolite values with an LCModel fit of a Cramér-Rao lower bound (CRLB) above 20% were excluded. Water T1 and T2 values were chosen as 2200 ms and 25 ms for 9.4T magnetic field [6]. Appropriate water attenuation correction was manually defined in the LCModel. The following equation was used to calculate the “real” metabolite concentration [M]real:

$${\left[M\right]}_{real}={{\left[M\right]}_{LCModel}/CF=[M]}_{LCModel}*\frac{N_{usup}}{N_{sup}}*\frac{{Gain}_{usup}}{{Gain}_{sup}}*\frac{{ATTH2O}_{real}}{{ATTH2O}_{default}}$$

In the above equation, CF is the correction factor; [M]_LCModel_ is the concentration value calculated from the LCModel; N_usup_ and N_sup_ are the numbers of signal averages in water-unsuppressed and suppressed signal; Gain_usup_ and Gain_sup_ are the receiver gains used for acquiring unsuppressed water signals and suppressed signals. Both the signal averages and receiver gains were determined from the scanning protocol we used. ATTH_2_O_default_ equals 0.7, a default attenuation correction factor assigned by the LC-Model. ATTH_2_O_real_ is defined as

$${ATTH2O}_{real}=e^{\left(-\frac{TE}{T_{2}}\right)}*e^{\left(1-\frac{TR}{T_{1}}\right)}$$

In our study, the calculated ATTH_2_O_real_ was 0.52, and total time for MRS was 15 min.

### Immunohistochemistry and Quantification of Htt Aggregates

Mice were anesthetized and perfused transcardially with phosphate-buffered saline (PBS) followed by 4% paraformaldehyde. Brains were post-fixed overnight followed by immersion in 30% sucrose for 24 h. Coronal brain sections (40 μm) were cut on a cryostat. Sections were stained with primary antibodies, including NeuN (1:300, Millipore, USA), EM48 (MAB5347, anti-huntingtin, 1:200, Merck Millipore, USA). Briefly, the sections were washed three times with PBS for 10 min each time, then permeabilized by incubating with 0.3% Triton X-100 for 5 min, followed by incubation with blocking solution containing 5% donkey serum, 3% goat serum and 0.3% Triton X-100 for 1 h. Then the sections were incubated with primary antibody at 4°C overnight. After three washings with PBS, the sections were incubated with fluorescence-labeled secondary antibody for 2 h at room temperature, and then washed 3 times with PBS. Sections were mounted onto superfrost slides (Fisher Scientific, Pittsburgh, PA, USA) dried and then covered with anti-fade mounting solution. Fluorescence images were acquired with a CCD camera attached to a fluorescence microscope (Zeiss). The percentage of neurons (Neu N positive cells) with nuclear mutant Htt aggregates (EM48-positive) was quantified with a 40 × magnification in the cerebral cortex and striatum of 6 and 12 month-old zQ175 mice. We counted the cells in eight microscopic fields for each brain region in each brain section.

### Western Blotting

Striatal tissue samples were homogenized in a buffer containing 50 mM Tris-HCl, pH 8.0, 150 mM NaCl, 0.1% (w/v) SDS, 1.0% NP-40, 0.5% sodium deoxycholate, and 1% (v/v) protease inhibitor mixture. For SDS PAGE, 30–50 μg of proteins were separated in a 4–20% gradient gel and transferred to a nitrocellulose membrane. The membrane was blotted with the following primary antibodies: anti-DARPP32 (Cell Signaling, rabbit polyclonal antibody, 1:1000), anti-C1qC (LifeSpan Bioscience, rabbit polyclonal antibody, 1:1000), anti-PSD95 (Thermo Scientific Pierce, mouse monoclonal antibody, 1:1000) and mouse anti-β-actin (Sigma, mouse monoclonal antibody, 1:5000). After incubation with HRP-conjugated secondary antibodies, the bound antibodies were visualized by chemiluminescence. The intensity of the Western blot bands was quantified by Image J software.

### Statistical Analysis

Data are expressed as mean ± SEM or SD as indicated in each figure. Repeated two-way (Age and Genotype) *ANOVA* was used for longitudinal body weight data, behavioral data and structural MRI data analysis. Student's *t*-test was used for other measures between WT and zQ175 groups with significance level set at *p* < 0.05.

## Results

### Body Weight Loss and Motor Dysfunction

**Fig 1:**
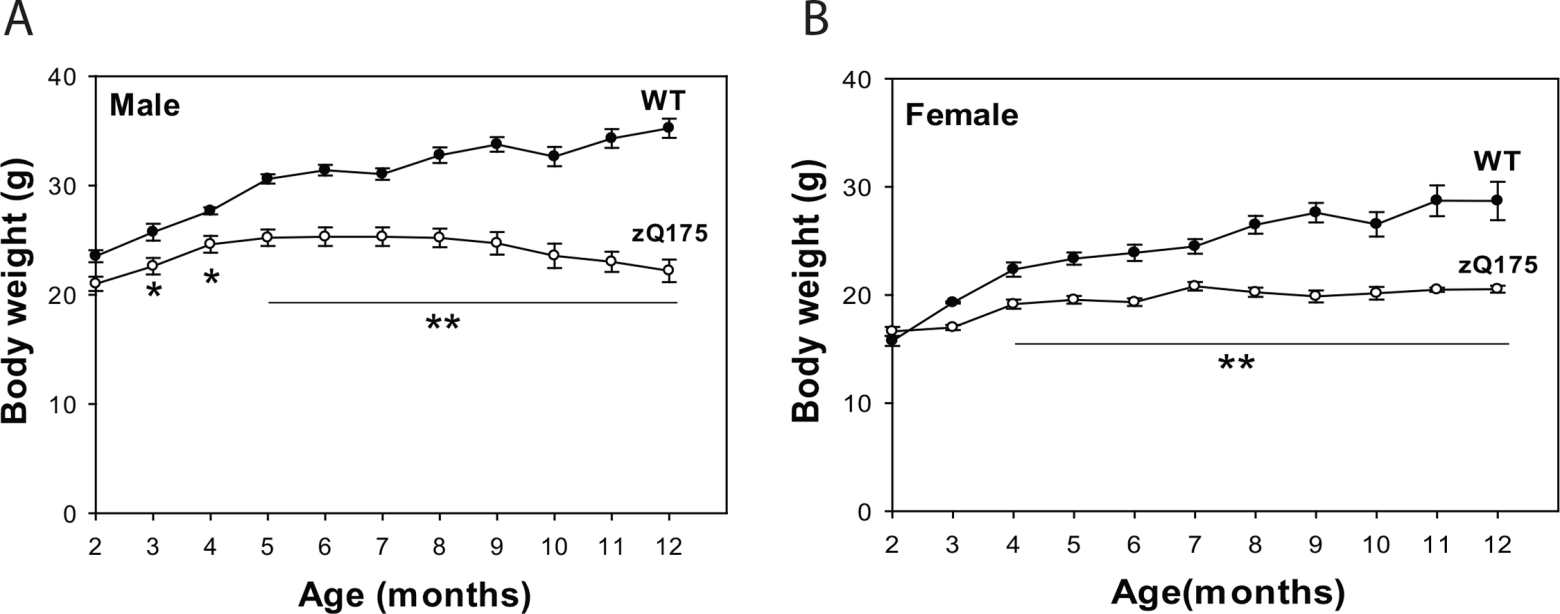
zQ175 mice exhibited lower body weight. Body weight (mean ± SD) of homozygous zQ175 mice and their age, gender-matched littermates was recorded weekly. *p<0.05 and **p<0.001 versus age-matched wild type (WT) mice by repeated two-way (age and genotype) *ANOVA*.To assess motor function of zQ175 mice, we conducted balance beam tests, accelerating rotarod test, and open field locomotor activity test with 3-, 6-, 9-, and 12-month-old mice. The zQ175 mice exhibited motor deficits indicated by longer time spent to cross the beam (**Fig 2A and 2B**), and reduced central activity and rear frequency at indicated ages (**Fig 2C–2E**). The motor deficits were progressive in zQ175 mice. The accelerating rotarod test did not show significant differences between zQ175 mice and controls until 12 months of age (**Fig 2F**).

Homozygous zQ175 mice displayed significantly lower body weight than did their WT littermate controls. Male mice exhibited significantly lower body weight from 3 months of age (**Fig 1A**), genotype main effect, F(1,1901) = 47.37, *p*<0.001; age main effect, F(10,812) = 74.12, *p*<0.001; Genotype × age interaction, F(10,481) = 43.94, *p*<0.001. Female mice showed significantly lower body weight from 4 months of age (**Fig 1B**), genotype main effect, F(1,965) = 50.86, *p*<0.001; age main effect, F(10,1336) = 105.90, *p*<0.001; Genotype × Age interaction, F(10,424.287) = 33.62, *p*<0.001. Loss of body weight is also a characteristics of HD patients [7]. This effect on body weight was not seen in heterozygous zQ175 until later ages [2].

**Fig 2:**
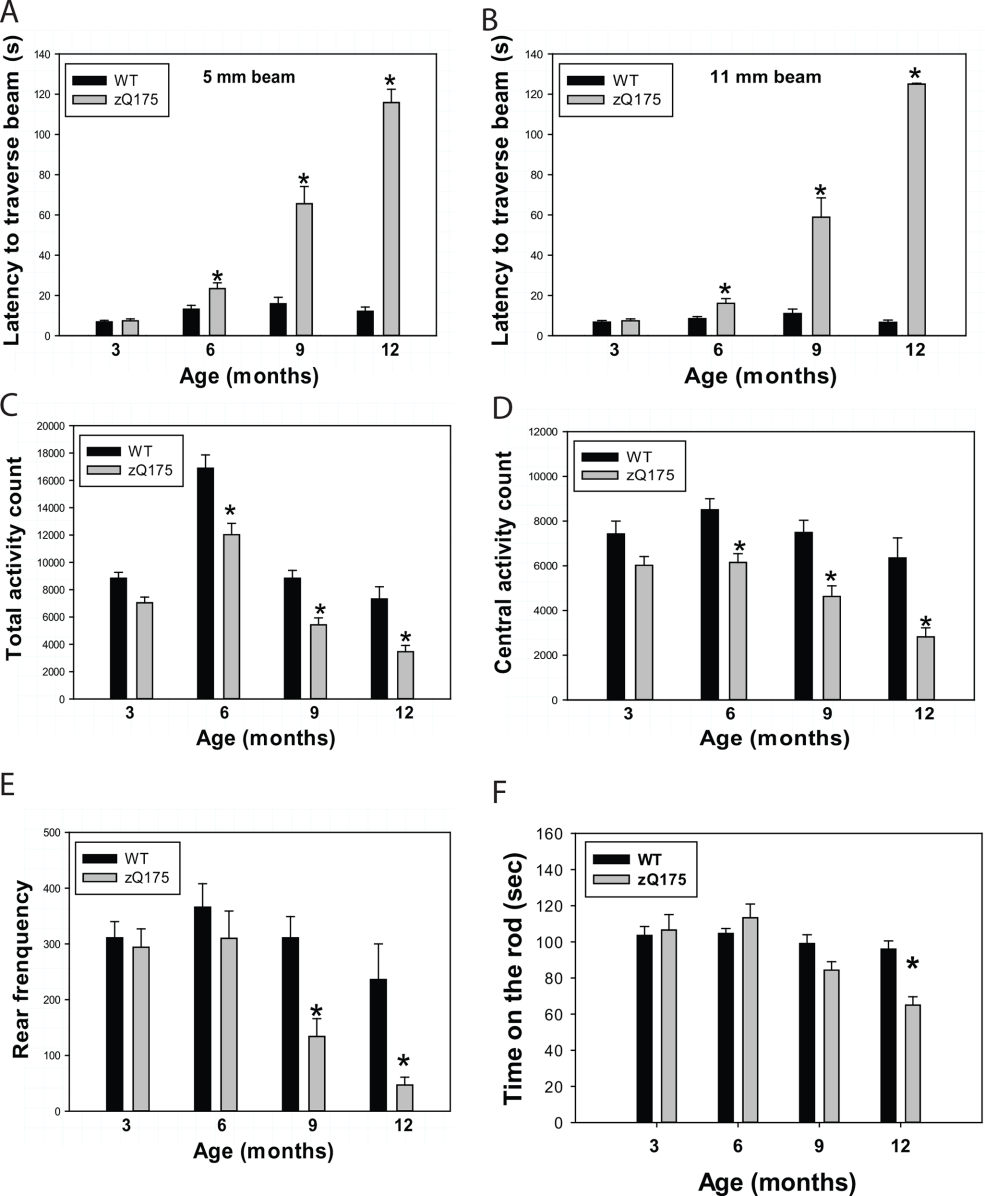
zQ175 mice displayed progressive motor deficits on balance beam and decreased locomotor activity in the Open Field chambers. (**A,B**) Mice were trained and tested on a 5 mm balance beam (A) or 11 mm beam (B), and latency to cross the beam was recorded. n = 16, 8 male and 8 female homozygous zQ175 mice. *p<0.05 versus age matched wild type (WT) mice by repeated two-way (Age and Genotype) *ANOVA*. (**C-E)** Locomotor activity was assessed in the Open Field apparatus; total activity (C), central activity (D), and rearing frequency (E) were recorded automatically during a 1 h testing period. n = 16, 8 males and 8 females. *p<0.05 versus age-matched wild type (WT) mice by one-way *ANOVA* with Fisher’s *posthoc* tests. (**F**) Time staying on the rotarod was recorded and averaged among three trials. n = 16, 8 males and 8 females. *p<0.05 versus age-matched wild type (WT) mice by one-way *ANOVA* with Fisher’s *posthoc* tests.

### Brain Atrophy Is Detected by *In Vivo* Structural MRI

**Fig 3:**
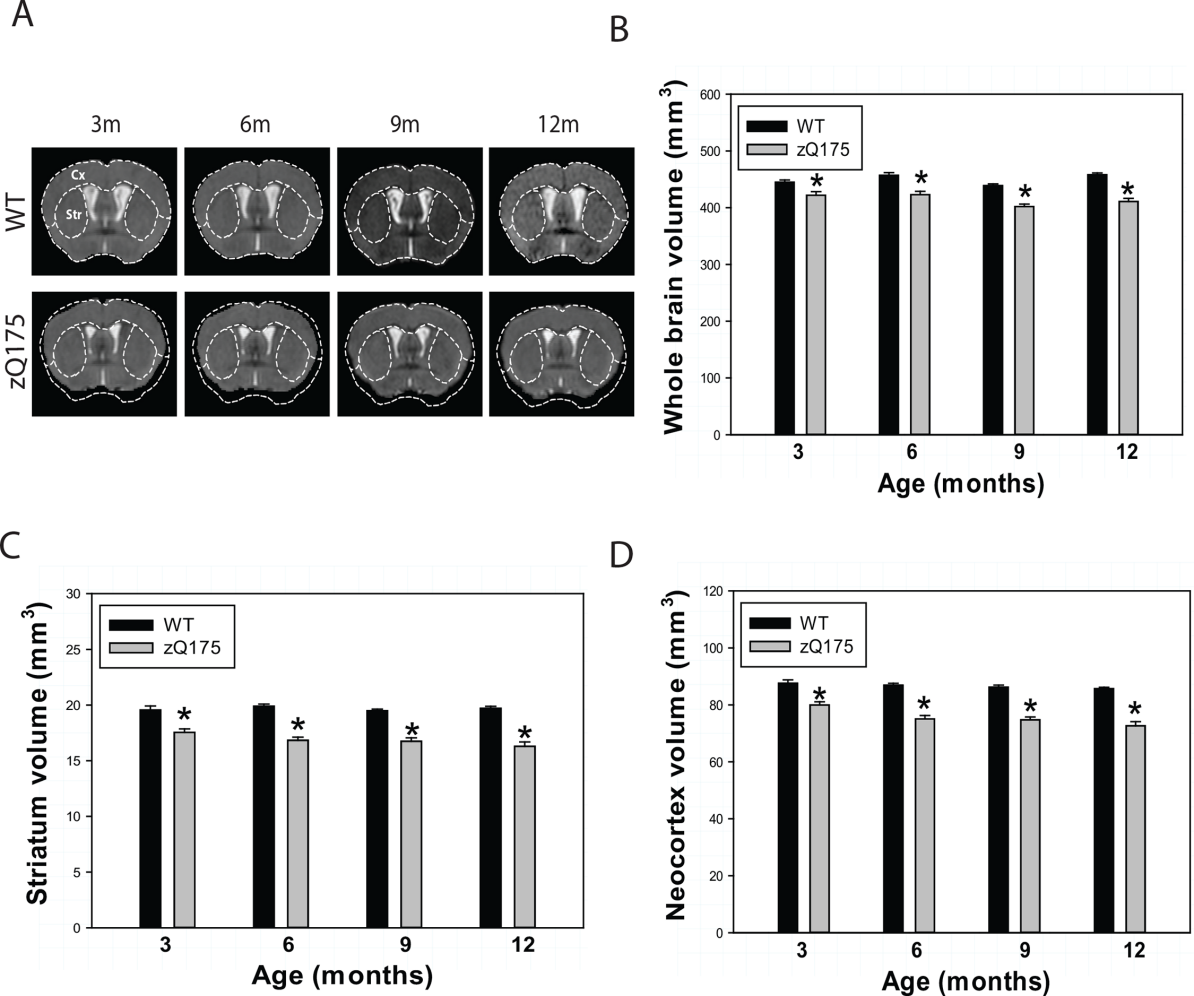
MRI detected significant brain atrophy, particularly in the striatum and neocortex and white matter abnormality in zQ175 mice. **(A)** Representative T2-weighted coronal MRI images from mice at indicated genptype. Note that the whole brain atrophy and regional brain atrophy in neocortex (Cx) and striatum (Str) in zQ175 mice. (**B-D**) *In vivo* longitudinal MRI quantification of volumes of whole brain (B), striatum (C), and neocortex (D) at indicated ages. n = 16 (8 male and 8 female homozygous zQ175 mice). *p<0.05 compared to the values of age-matched control mice by one-way ANOVA with Fisher’s *posthoc* tests.

Although mutant *htt* is expressed ubiquitously, the neuropathology in HD is selective, in which robust atrophy is seen in the striatum and to some extent in the cortex, and extends to other brain regions with disease progression. In order to determine whether the zQ175 mice also have selective brain atrophy, we performed *in vivo* longitudinal structural MRI whole brain scans from 3 months to 12 months of age. Homozygous zQ175 mice displayed significant atrophy in the striatum and neocortex at an early age (**Fig 3A–3C**), even before motor deficits were detected on the balance beam or open field activity chamber. This is a similar phenomenon as shown in HD patients who often displayed brain atrophy in MRI measures decades before clinical symptoms.

### Altered Striatal Metabolites Detected by MRS

**Fig 4:**
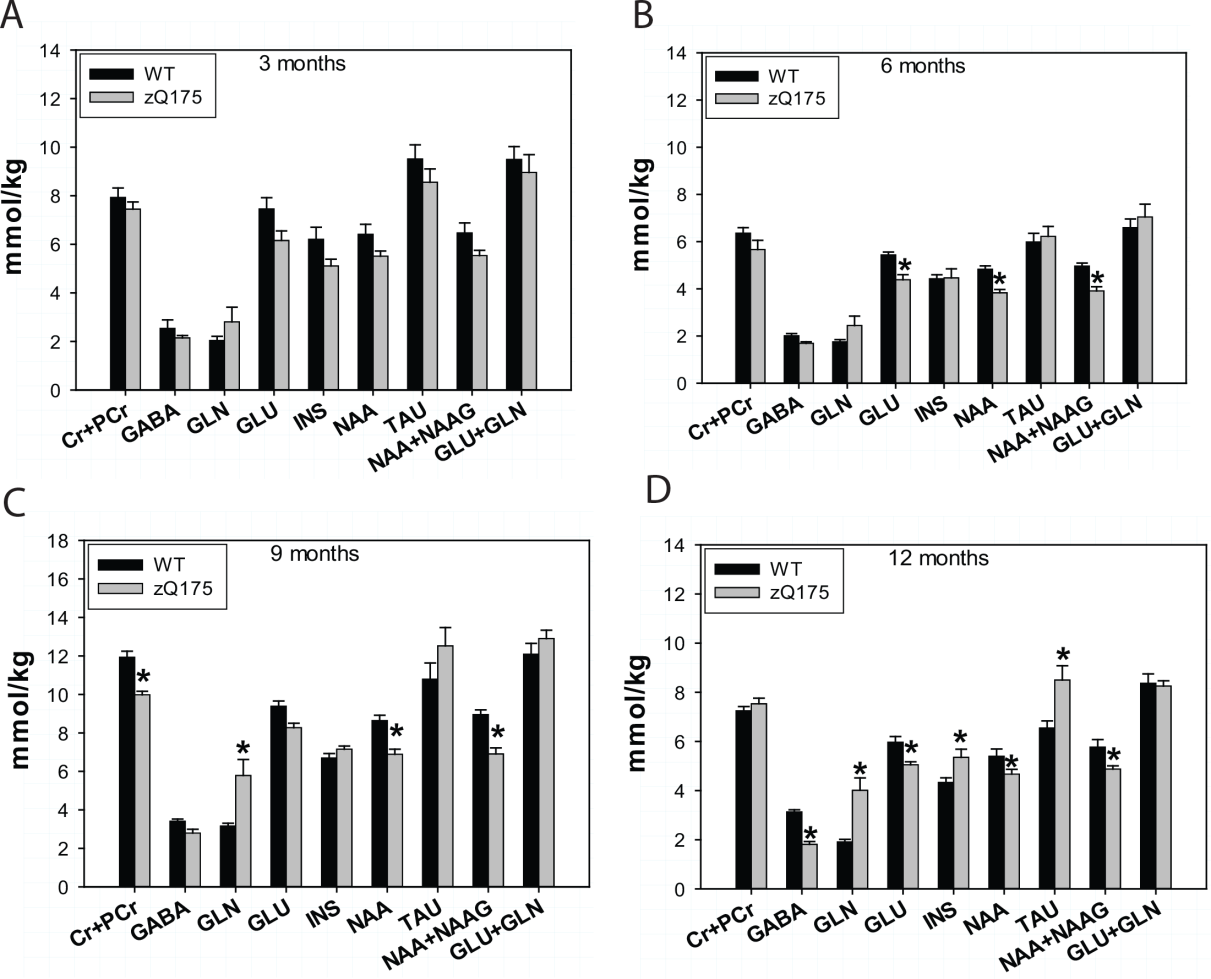
zQ175 mice show abnormal brain metabolites in the striatum measured by (1)H MRS. Metabolite values with an LC Model fit of a Cramér-Rao lower bound (CRLB) above 20% were excluded. Following metabolites, including creatine (Cr), phosphocreatine (PCr), gamma-aminobutyric acid (GABA), glutamine (GLN), glutamate (GLU), inositol (INS), N-acetylaspartate (NAA), taurine (TAU), N-acetyl-L-aspartyl-L-glutamic acid (NAAG), were measured in the striatum of mice. Quantification of brain metabolites in the mouse striatum in indicated genotypes and ages in both male and female homozygous zQ175 mice. n = 8 (4 males and 4 females). *p<0.05 compared to the values of wild type (WT) mice by standard Student’s *t*-tests. The spectrum was obtained by the standard PRESS sequence (TE = 15 ms, TR = 5 s, 256 averages). After an initial scout image, a 4 mm x 4 mm voxel was placed in the mouse striatum. First and second order shim coil currents were adjusted by using FASTMAP, the line after which width of water was approximately 15 Hz. Adjustment of the LC Model quantification process was made with respect to the water relaxation time and concentration.

Impaired energy production and increased energy demand are evident in HD [8]. Magnetic resonance spectroscopy (MRS) allows noninvasive measurements of the concentrations of brain metabolites, several of which are involved in brain energy metabolism. To determine whether brain metabolites are altered in zQ175 mice, we employed proton MRS (^1^H MRS) to measure *in vivo* neurochemical profiles together with principal component analysis. We were able to reliably measure nine metabolites in the mouse striatum. We started assessing metabolite levels in the striatum of 3-month-old mice, and measured the metabolite concentrations every 3 months longitudinally thereafter. At 3 months, there were no significant differences in the metabolites between zQ175 mice and WT mice (**Fig 4A**). By 6 months of age, zQ175 mice displayed lower levels of *N*-acetylaspartate (NAA) and glutamate (GLU) in striatum than did WT mice (**Fig 4B**). At 9 months of age, glutamine (GLN) levels were significantly increased and creatine (CR) plus phospho-creatine (PCr) levels were decreased in the zQ175 mouse striatum (**Fig 4C**). At 12 months of age, most metabolites were significantly altered in the striatum of zQ175 mice, including decreased levels of gamma-aminobutyric acid (GABA), GLU, NAA, and increased GLN and taurine (TAU) levels (**Fig 4D**).

### Mutant Huntingtin Aggregation

**Fig 5:**
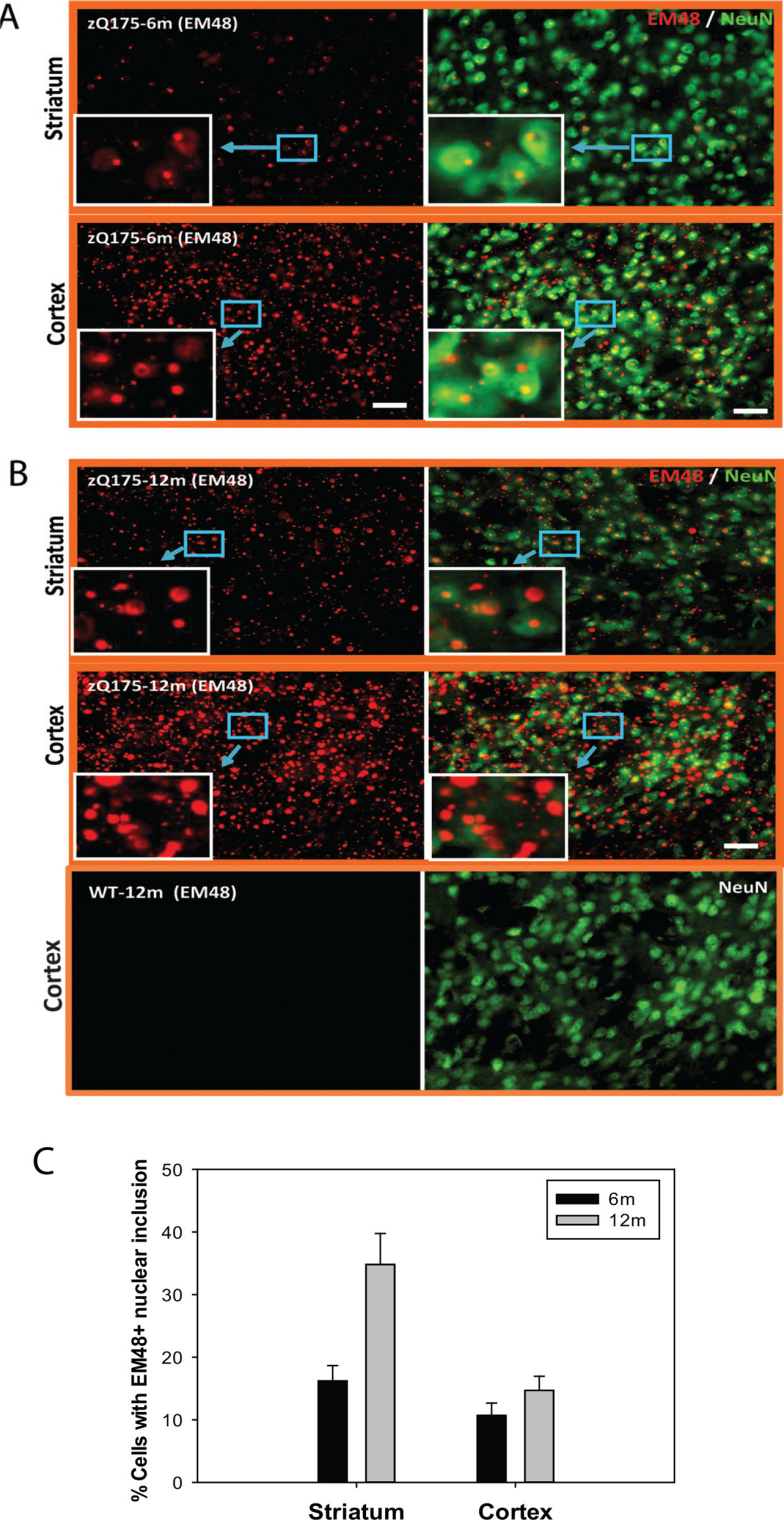
Mutant huntingtin aggregates are detected in zQ175 mouse brain. Immunofluorescent staining was performed in the brain sections of zQ175 mice at indicated ages- 6 months (A) and 12 months (B). Representative pictures were taken in both striatum and cortex area. EM48-positive mutant huntingtin aggregates (red fluorescent signal) and neuronal marker NeuN (green color) were labeled. Scale bar = 200 μm. (C) Neurons with nuclear EM 48 positive mutant Htt aggregates were quantified in both striatum and cortex regions at 6 month and 12 month old mice. Percentage of neurons with nuclear mutant Htt aggregates was presented. n = 3.

Previous studies of mutant Htt aggregation in mouse models expressing full-length mutant *Htt* revealed selective nuclear accumulation of aggregated mutant protein in the striatum and cortex [9–14]. We detected widely distributed mutant Htt aggregates by EM48 antibody in zQ175 mouse brain. The numbers of neurons with nucear EM48 positive Htt aggregates increased with age in both the striatum and cortex of zQ175 mice (**Fig 5**).

### Altered Protein Markers in the Striatum

**Fig 6:**
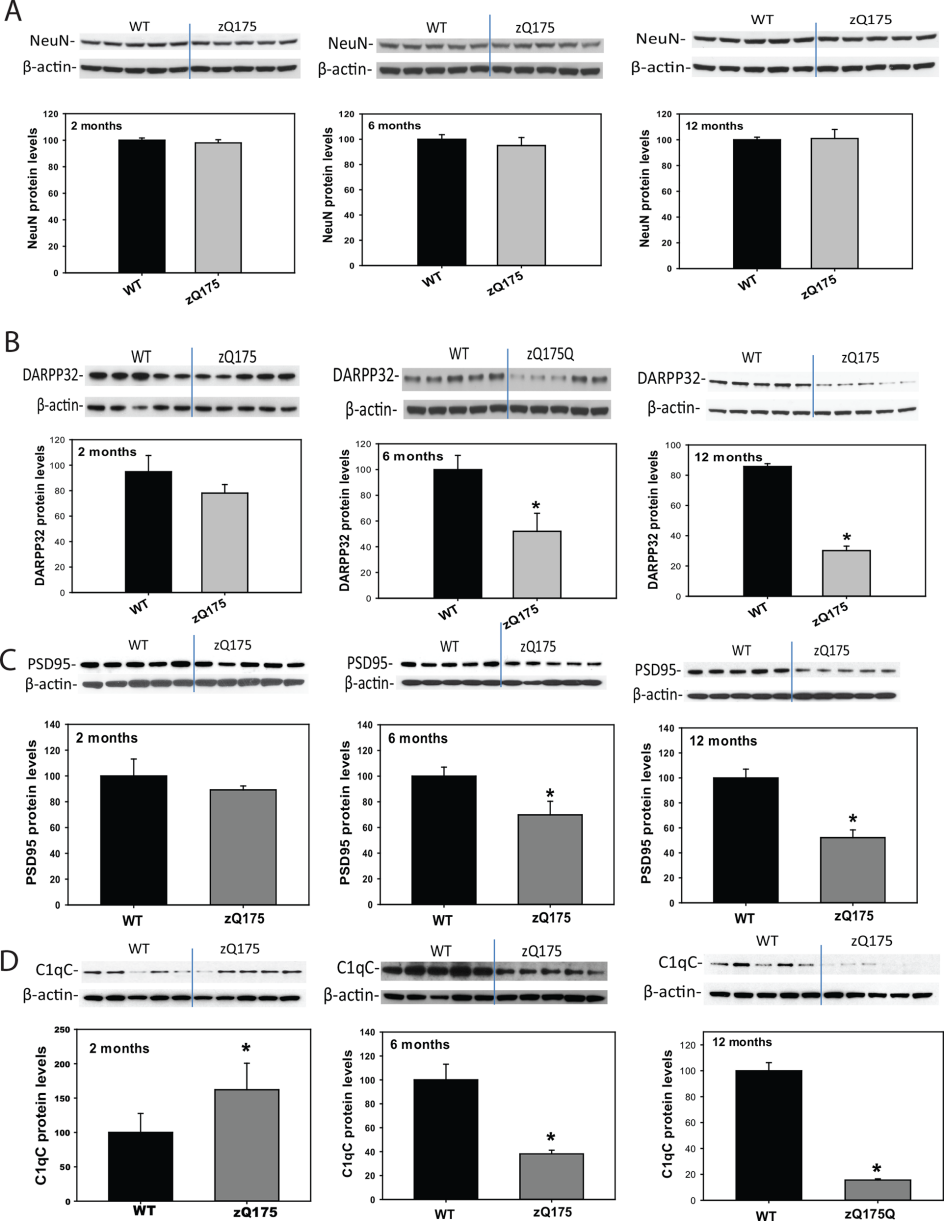
zQ175 mice exhibited significant loss of medium spiny neuronal marker, postsynaptic protein, and altered complement components C1qC in the striatum. Protein levels of NeuN (A), DARPP32 (B), PSD95 (C), and C1qC (D) were assessed in the striatal tissue at indicated ages in zQ175 mice by Western blotting. n = 5. *p<0.05 compared to the values of age-matched wild type (WT) mice by standard Student’s *t*-tests.

To identify molecular markers in the brain of this mouse model, we evaluated general neuronal markers as well as spiny medium neuronal markers, and found that NeuN, a general neuronal marker, did not distinguish between zQ175 mice and controls (**Fig 6A**), while the protein levels of DARPP32, the medium spiny neuronal marker, were significantly and progressively reduced in the striatum of zQ175 mice (**Fig 6B**); these results suggest that decreased DARPP32 protein levels may be due to selective neuronal dysfunction rather than general neurodegeneration. We also detected a decrease in the postsynaptic marker protein PSD95 in zQ175 mouse striatum (**Fig 6C**). Interestingly, the inflammatory factor C1qC levels were increased at the early disease stage in zQ175 mice, and then reduced significantly in the later ages (**Fig 6D**).

## Discussion

Our present study confirmed that the zQ175 mice have a much more robust phenotype than do the original HdhQ140 mice. By replacing the short CAG repeat of the mouse *Hdh* with an expanded repeat of human *Htt* exon-1, zQ175 KI mice represent genetic replicas of human HD more closely than transgenic models in which mutant *Htt* is overexpressed. We found that both male and female mice exhibited key features of HD, such as selective brain atrophy, motor deficits, altered brain metabolites, mutant Htt protein aggregation. Dysregulated selective neuronal marker proteins were evident in zQ175 mice.

Notably, the CAG repeat size of mutant *Htt* is much shorter in HD patients than that resulting in phenotypes in this model. The perturbation induced by mutant *Htt* in the mouse model may not equally recapitulate the human HD abnormality. Inherent differences between humans and mice must be considered in the search for efficacious treatments for HD. There is almost complete striatal degeneration in HD patients with a 40–50 CAG repeats, but mice expressing full-length mutant *Htt* with much longer CAG repeats (more than 100 CAG repeats in YACHD, KI lines-Q140, 150, 175, 200) do not show dramatic degeneration. Consistent with this observation in human HD, mutant *Htt* carriers have a smaller intracranial adult brain volume before the onset of the disease than do controls, probably reflecting abnormal development [15]; indeed, the brain atrophy was evident in zQ175 mice, even before the motor deficits were detected, suggesting that abnormal brain development may also have contributed to the brain volume change, particularly in the early disease phase, similar to human HD.

Hypoactivity, as measured by decreased horizontal activity in the open field chamber, was seen in zQ175 mice. This hypolocomotor activity is similar to that described previously [2]. Brain atrophy has been detected by MRI in other full-length HD mouse models, such as YAC128 mice [16, 17] and HdhQ250 KI mice [18]. zQ175 mice demonstrated selective atrophy in the striatum and neocortex resembling neuropathology in human HD. These volumetric changes were detected by noninvasive structural MRI, which provides digitized data with full brain coverage, free from distortions due to embedding and sectioning. Furthermore, such MRI-based volumetric determinations are routinely used in humans, making preclinical mouse studies directly scalable to human clinical trials [19, 20]. Development of a complete natural history of brain pathological changes by longitudinal imaging during preclinical trials would considerably increase the power to detect therapeutic efficacy compared to a single assessment.

Alterations in brain metabolism indicate HD progression. N-Acetylaspartate (NAA) was reduced markedly in both presymptomatic and symptomatic HD patients, and reduced NAA correlated highly with the motor score of the Unified Huntington's Disease Rating Scale [21]. Other studies also suggested disturbed brain metabolites in HD patients [22–24] and HD mice [25, 26], indicating that altered brain metabolite levels may serve as alternative biomarkers in clinical trials. We detected significantly altered striatal metabolites in 6, 9, and 12 months old zQ175 mice, reminiscent of some changes in human HD brain [27, 28], such as decreased NAA and GLU levels and increased taurine. The disturbed metabolites are not simply due to neuronal loss, as some metabolites (NAA, GLU) decreased, and others (taurine) increased in HD brain. Altered metabolites in the zQ175 mouse brain are also similar to the results reported previously [3], suggesting that brain metabolite changes may serve as alternative biomarkers in using these HD models. We do not know why the changes of metabolites were only detectable in later ages of zQ175 mice, but other studies in R6/2 mice and HdhQ111 KI mice suggest a potential compensatory response that may maintain energetic homeostasis from early ages through some manifest stages [29, 30].

Aggregation of mutant huntingtin protein is a pathological hallmark of HD [31–33]. In adult-onset HD patients, mutant huntingtin aggregation patterns predominantly consist of large aggregates in the neuropil, with only a small percentage of aggregates present in the nucleus. Interestingly, this nuclear versus cytoplasmic distribution of mutant huntingtin aggregates in the cortex is opposite to that in juvenile-onset HD patients, in whom nuclear inclusions are the predominant species [31]. Additionally, more large aggregates are observed in the cortex of both juvenile- and adult-onset HD brains when compared with those in the striatum [31, 32]. Mutant Htt aggregates detected in the striatum and cortex of zQ175 mice are localized in both nucleus and neuropil, with small percentage of neurons with nuclear aggregates as indicated in Fig 5C.

Altered gene expression and protein levels in the striatum are reported in both human HD and mouse models, in particular, decreased levels of medium spiny neuronal marker DARPP32 [10, 25, 34–44] and postsynaptic protein PSD95 were reported in other HD models [45], including zQ175 mice [2]. We show here that levels of DARPP32 and PSD95 were decreased in zQ175 mice, suggesting that selective neurodegeneration and synaptic dysfunction exist in this model; while complement component protein C1qC was increased at an early age, indicating that inflammation may be involved in early HD pathogenesis in the zQ175 mouse brain.

Taken together, the robust phenotype of zQ175 mice, resembling key features of HD, and the relatively small variability of several measures in this mouse line provides a novel model for identifying therapeutics. Our findings further confirmed the value of zQ175 model and shed light on the understanding of HD pathogenesis and the use of appropriate measures for different disease phases.
